# Co-prescription of medication for bipolar disorder and diabetes mellitus: a nationwide population-based study with focus on gender differences

**DOI:** 10.1186/1741-7015-10-148

**Published:** 2012-11-27

**Authors:** Gjertrud Svendal, Ole Bernt Fasmer, Anders Engeland, Michael Berk, Anders Lund

**Affiliations:** 1 Department of Clinical Medicine, Section for Psychiatry, University of Bergen, Sandviken Hospital, Sandviksleitet 1, N-5035 Bergen, Norway; 2 MoodNet Research Group, Haukeland University Hospital, Sandviken Hospital, Sandviksleitet 1, N-5035 Bergen, Norway; 3 Department of Pharmacoepidemiology, Division of Epidemiology, Norwegian Institute of Public Health, PO Box 4404 Nydalen, N-0403 Oslo, Norway; 4 Department of Public Health and Primary Health Care, University of Bergen, PO Box 7804, N-5020 Bergen, Norway; 5 Deakin University, School of Medicine, 1 Gheringhap Street, Geelong VIC 3220, Australia; 6 The University of Melbourne, Department of Psychiatry, Level 1, North Block Main Building Royal Melbourne Hospital, Parkville VIC 3050, Australia; 7 Orygen Youth Health Research Centre, Centre for Youth Mental Health, 35 Poplar Road, Parkville VIC 3052, Australia; 8 Florey Institute of Neuroscience and Mental Health, Level 3, Alan Gilbert Building, 161 Barry Street, University of Melbourne, Parkville VIC 3010,, Australia

## Abstract

**Background:**

Studies have shown a correlation between bipolar disorder and diabetes mellitus. It is unclear if this correlation is a part of common pathophysiological pathways, or if medication for bipolar disorder has negative effects on blood sugar regulation.

**Methods:**

The Norwegian prescription database was analyzed. Prescriptions for lithium, lamotrigine, carbamazepine and valproate were used as proxies for bipolar disorder. Prescriptions for insulin and oral anti-diabetic agents were used as proxies for diabetes mellitus. We explored the association between medication for bipolar disorder and diabetes medication by logistic regression

**Results:**

We found a strong association between concomitant use of medication to treat diabetes mellitus and mood stabilizers for the treatment of bipolar disorder. Females had a 30% higher risk compared to men of being treated for both disorders. Persons using oral anti-diabetic agents had higher odds of receiving valproate than either lithium or lamotrigine. Use of insulin as monotherapy seemed to have lower odds than oral anti-diabetic agents of co-prescription of mood stabilizers, compared to the general population.

**Conclusions:**

This study showed a strong association between the use of mood stabilizers and anti-diabetic agents. The association was stronger among women than men.

## Background

The comorbidity between diabetes mellitus and bipolar disorder has been explored in several studies, with the estimated prevalence varying from about two to five times higher than in the general population [1-4]. Most studies have had a small number of participants, and a majority have investigated the comorbidity in hospitalized patients, where selection bias may color the results. Few studies have examined whole population samples and no studies, to our knowledge, have looked at gender differences with regard to comorbidity between bipolar disorder and diabetes mellitus. The European Psychiatric Association emphasizes the need to focus on diabetic comorbidity in patients with severe mental illness [5].

In this cross-sectional study, the Norwegian prescription database (NorPD), which encompassed prescriptions from the total Norwegian population in 2006, was analyzed. Using prescriptions as a proxy for clinical diagnoses we aimed to (I) investigate the comorbidity between diabetes mellitus and bipolar disorder, (II) explore the effect of gender, (III) look at differences between insulin and non-insulin dependent diabetes, and (IV) between different mood stabilizing drugs.

## Methods

Data from the NorPD for 2006 were explored. NorPD contains information on all prescribed drugs, reimbursed or not, dispensed at pharmacies to individual patients treated in ambulatory care. Data on use in institutionalized patients in nursing homes and hospitals are also collected, but these figures are only registered at an institutional level. Therefore, drugs dispensed in institutions are not included in our study. All drugs for chronic diseases in Norway are covered by the public health care system. This method is extensively described in articles analyzing data from the database [6-9]. The medications, which were the object of analysis in this study, were mood stabilizers for the treatment of bipolar disorder, and insulin and oral anti-diabetic agents for the treatment of diabetes mellitus. The data were analyzed to ascertain co-prescription of treatments for bipolar disorder and diabetes mellitus.

### Measurements

The medications are grouped according to the Anatomical Therapeutic Chemical (ATC) classification. We included patients with prescriptions for N03AG01 (valproate), N03AX09 (lamotrigine), N03AF01 (carbamazepine), N05AN01 (lithium), A10A (insulin and insulin analogues) and A10B (oral anti-diabetics) (Table 1). In the analyses we have also included antipsychotic medication as a possible confounder and stratifier. Chlorpromazine (N05AA01), levomepromazine (N05AA02), perphenazine (N05AB03), prochlorperazine (N05AB04), haloperidol (N05AD01), flupenthixsol (N05AF01), chlorprothixene (N05AF03) and zuclopenthixol (N05AF05) were catagorized as first generation antipsychotic medications. Sertindole (N05AE03), ziprasidone (N05AE04), clozapine (N05AH02), olanzapine (N05AH03), quetiapine (N05AH04), risperidone (N05AX08) and aripiprazole (N05AX12) were categorized as second generation antipsychotics. In this study, only valproate, lamotrigine and carbamazepine used as medication for bipolar disorder are included, excluding those people using these medications that were coded as being for the treatment of epilepsy. This information is included in the dataset.

**Table 1:** Table showing drugs used in the analyses

Anatomical Therapeutic Chemical Classification System (ATC-nr)	Chemical therapeutic level	Number of individuals with at least one prescription of medication	Percentages of persons receiving treatment
Bipolar disorder		17,007	0.6
N03AG01	Valproate	3,728	0.1
N03AX09	Lamotrigine	6,501	0.2
N03AF01	Carbamazepine	2,049	0.1
N05AN01	Lithium	6,538	0.2

Diabetes mellitus		77,669	2.7
A10A	Insulin or insulin analogs	32,005	1.1
A10B	Oral antidiabetic agents	55,665	1.9

The drugs are categorized by both their ATC number and their chemical therapeutical name. The number of persons receiving the medications, are aged 20 *to *69.

### Statistical analyses

While the data file obtained from the prescription database included the total Norwegian population of 2006, we chose to only include persons aged 20 to 69 years (*n *= 2,929,065, males *n *= 1,480,648 and females *n *= 1,448,417). Odds ratios (OR) and 95% confidence intervals (CI) were obtained from binary logistic regression to explore the association between the types of prescriptions. Odds ratios were used as approximations of relative risk. All analyses were performed using the Statistical Package for Social Sciences (SPSS) version 19.0 (IBM SPSS Statistics 19, SPSS inc., an IBM Co., Somers, NY, USA).

### Ethical approval

The patients in the prescription database had an encrypted unique individual identity-number. Because of this, no regional ethics committee (IRB) approval was required for this study.

## Results

In 2006, a total of 77,669 persons between the ages of 20 and 69 received either an oral anti-diabetic agent (Table 1), insulin or both in Norway (2.7% of the population). Insulin as a monotherapy for diabetes mellitus was received by 22,004 (0.8%) persons, and oral anti-diabetic agents as monotherapy were received by 45,664 (1.6%) persons. A total of 10,001 (0.3%) used both insulin and oral anti-diabetics agents. Treatment for bipolar disorder (valproate, lithium, carbamazepine or lamotrigine) was received by 17,007 persons (0.6%). Males more frequently received treatment for diabetes mellitus than females (males = 3.0%, females = 2.3%), while for bipolar disorder females more frequently received treatment (males = 0.5%, females = 0.7%). The proportion of persons receiving prescriptions increased substantially with age for oral anti-diabetic agents, while a lower proportional increase was observed for insulin.

A total of 900 persons who received treatment for bipolar disorder also received treatment for diabetes mellitus, this is 2.1 (CI 95%: 1.9, 2.2) times more frequent than the rest of the population. Adjusting for sex and age, the odds ratio was 2.0 (CI 95%: 1.8, 2.1). The odds for persons receiving insulin in monotherapy to also receive treatment for bipolar disorder was 1.7 (CI 95%: 1.5, 2.0) times higher than in the general population. Persons receiving oral anti-diabetic agents in monotherapy had an odds ratio for also receiving bipolar medication that was 2.2 (CI 95%: 2.0, 2.3) times higher than in the general population.

When adjusting for the use of antipsychotic medications as a possible confounder, the odds for receiving mood stabilizers and medication for diabetes mellitus was 1.3 (CI 95%: 1.2, 1.4), adjusted for age and gender. Excluding all persons receiving antipsychotic medication, both first and second generation, the odds for receiving medication for diabetes mellitus and bipolar disorder was 1.6 (CI 95%: 1.4, 1.7) compared to the general population, adjusted for gender and age.

In Table 2, the odds of receiving at least one of the mood stabilizers for persons using insulin or oral anti-diabetic agents was compared to the odds for the rest of the population. The OR was higher in females than in males for all combinations. Females had an OR of 1.3 (1.3, 1.4) compared to men of being treated with both mood stabilizers and anti-diabetic drugs, compared to the general population.

**Table 2:** Odds ratios for co-prescription of anti-diabetes agents and mood stabilizers.

					Total	
	**Male**		**Female**			
			
**Drugs**	**OR**	**CI (95%)**	**OR**	**CI (95%)**	**OR**	**Cl (95%)**

Lamotrigine and insulin	1.8	(1.3, 2.5)	1.8	(1.3, 2.4)	1.7	(1.3, 2.1)
Carbamazepine and insulin	2.0	(1.3, 3.2)	3.0	(1.9, 4.8)	2.4	(1.8, 3.4)
Lithium and insulin	1.4	(1.0, 1.9)	1.6	(1.1, 2.2)	1.4	(1.1, 1.8)
Valproate and insulin	1.6	(1.1, 2.3)	2.6	(1.8, 3.7)	2.0	(1.5, 2.6)

Lamotrigine and oral antidiabetics	1.3	(1.0, 1.7)	1.8	(1.5, 2.3)	1.6	(1.3, 1.9)
Carbamazepine and oral antidiabetics	1.8	(1.3, 2.5)	2.6	(1.8, 3.6)	2.1	(1.7, 2.7)
Lithium and oral antidiabetics	1.5	(1.2, 1.8)	2.6	(2.2, 3.0)	1.9	(1.7, 2.2)
Valproate and oral antidiabetics	2.4	(1.9, 3.0)	3.0	(2.3, 3.7)	2.6	(2.2, 3.1)

Odds ratios, with 95% CI, for using different mood stabilizers, combined or in in monotherapy for persons using anti-diabetes agents in monotherapy compared to the non diabetic population (divided into genders).

There were some differences in OR between the drugs used in treatment for bipolar disorder, as seen in Table 2. Persons receiving oral anti-diabetic agents in monotherapy had an odds ratio of also receiving valproate combined or in monotherapy that was 2.6 (CI 95%: 2.2, 3.1) times higher than in the remainder of the population. This was significantly different from the odds for lamotrigine combined or in monotherapy and oral anti-diabetic agents in monotherapy, OR: 1.6 (CI 95%: 1.3, 1.9), and lithium and oral anti-diabetics, OR: 1.9 (1.7, 2.2), both compared to the remainder of the population.

## Discussion

Our main findings were: (I) a strong association between use of medication to treat diabetes mellitus and mood stabilizers for the treatment for bipolar disorder, and (II) females had a 30% higher risk compared to men of being treated simultaneously for both disorders. We also found (III) that persons using oral anti-diabetic agents had higher odds of receiving valproate than either lithium or lamotrigine. (IV) Persons receiving insulin in monotherapy seemed to have a lower odds ratio than persons receiving oral anti-diabetic agents of also receiving mood stabilizers, compared to the general population.

### Matching results

The present findings are in agreement with previous studies showing comorbidity between diabetes mellitus and bipolar disorder. Two studies have shown a prevalence of diabetes mellitus of 10% in a group of hospitalized bipolar patients [1,2]. A study [3] found a 26% prevalence of type II diabetes among patients with bipolar 1 disorder. Another study reported [10] a 11.7% prevalence of diabetes in the bipolar population, and that patients with comorbid diabetes mellitus and bipolar disorder had a 81% disability rate, compared to 30% in the non-diabetic bipolar population. None of the studies noted whether this comorbidity differed by gender, or if the patients were affected by medication use. Carney [11] investigated medical comorbidity in women and men with bipolar disorder. They found diabetes with complications to occur at increased odds (adjusted OR = 1.54) among 3,557 subjects with bipolar disorder, compared to the non-bipolar patients in the sample group. However, the odds of having uncomplicated diabetes was not significantly elevated compared to the control population. This finding may be related to the fact that individuals with psychiatric disorders tend to get poorer and more sporadic medical treatment than the general population. Carney *et al. *found that more woman (61%) than men (39%) were identified with bipolar disorder. However, they did not investigate whether comorbidity of bipolar disorder and diabetes mellitus was different for males and females. Fiedorowicz [4] found a higher prevalence of diabetes (30%) in 217 bipolar patients, compared to the general population (12.5%). In a small sample (women n = 25, men = 18), they found men to have a higher prevalence of diabetes mellitus (33%) than women (28%), but found no significant association between medication use and cardiovascular risk factors.

### Conflicting results

Kessing *et al. *[12] investigated whether some of the co-morbidity between diabetes and bipolar disorder could be the result of conditional probability, or Berkson's bias. Patients with osteoarthritis were compared to patients with bipolar disorder. They found a slightly increased rate of diabetes among people with bipolar disorder compared to those with osteoarthritis (aged between 45 and 80 years). However, the prevalence of diabetes mellitus may have been underestimated and the control group of osteoarthritis is not ideal [13], due to a not yet understood association between osteoarthritis and insulin [14-16]. For example, osteoarthritis and diabetes share an etiological role of inflammatory pathways, as do mood disorders [17].

### Possible mechanisms

Three of the four mood stabilizers analyzed in the present study (carbamazepine, valproate and lithium) all have the potential to increase weight and, therefore, may increase the risk of diabetes type II. Mood stabilizers may also increase risk of diabetes mellitus in other ways than weight gain. Insulin tends to be used more for type I diabetes, and oral hypoglycemic agents more for type II diabetes, and the linkage between diabetes and bipolar disorder is stronger for type II than for type I diabetes. Valproate and lithium both inhibit glycogen synthase kinase 3b [18,19], as does insulin.

The finding of the high diabetes prevalence in patients with bipolar disorder could indicate that those with this disorder are at higher risk for diabetes by virtue of a shared pathophysiology common to both disorders, such as inflammation [20,21]. There is a genetic risk factor in both diabetes mellitus and bipolar disorder [22,23].

Women tend to experience more side effects from bipolar treatment [24], particularly weight gain, which may be a factor explaining the increased OR we found in women compared to men.

### Strengths and limitations

The results are based on data from the total Norwegian population, and the risk of selection bias is low. Our sample was initially large; however, only 900 persons received medication for both bipolar disorder and diabetes mellitus. This number is nevertheless higher than in any other study we know. The lack of inclusion of data from hospitalized and institutionalized patients probably represents a small number, as few patients stay in the hospital throughout an entire year and the majority of the institutionalized patients are 70 years and older in nursing homes. Mood stabilizers are used less frequently by persons younger than 20 and were, therefore, excluded. Treatment with insulin is not indicated for any other disease than diabetes mellitus. Persons at risk for developing diabetes mellitus type II rarely use oral anti-diabetic agents. In Norway, metformin is rarely used prophylactically in psychiatric patients. Lithium is to some extent also used for treatment of unipolar depressed patients; excluding persons on antidepressants did not substantially change the OR.

Mood stabilizers might be used by patients diagnosed with other psychiatric diagnoses, such as schizophrenia, and this presents a possible confounder, as the prevalence of diabetes is increased in patients with schizophrenia [25]. We are not aware of data from Norway on the use of mood stabilizers in schizophrenia, and data from other countries vary widely [26,27] so it is difficult to know how important this confounder may be. Of probable greater importance is the ability of antipsychotics to induce diabetes [28]. However, when adjusting for the use of antipsychotics the odds of receiving medication for bipolar disorder and diabetes mellitus was still significantly elevated (1.3). When excluding all persons using any antipsychotic agents, the odds increased to 1.6. Antipsychotic medication can possibly account for some of the correlations we found, but not all. Ruzikova [10] found that patients with bipolar disorder had a greater chance of complicated diabetes mellitus. It is therefore possible that patients with severe bipolar disorder, receiving both mood stabilizers and antipsychotic treatment, may have a higher risk of also receiving medication for diabetes mellitus. This needs to be investigated further.

The prescription database indirectly measures the frequency of diabetes and bipolar disorder in the adult population, and our risk estimates may, therefore, be interpreted as a proxy of comorbidity between the diseases. The validity of this measure is likely high, as all registered prescriptions are based on a physician's examination, diagnostic decision, as well as the fact that the patients have collected their medication. The study does not include persons who either do not seek treatment, recognize their illness or for any other reason do not collect their medication. As such, the study may have under-estimated the true prevalence and, thus, comorbidity. The cost for medical treatment is to a large extent covered by the public health care system in Norway, and very few persons will fail to purchase medication for economic reasons.

## Conclusions

This study has demonstrated a significant co-prescription of drugs used to treat diabetes and bipolar disorder, which may reflect shared risk pathways, as well as a possible role of treatment. This has implications for both the understanding of neurobiology and management.

## Abbreviations

**ATC**: Anatomical Therapeutic Chemical; CI: confidence interval; NorPD: Norwegian Prescription Database; OR: odds ratio.

## Competing interests

GS has no conflicts of interest, including specific financial interests and relationships and affiliations relevant to the subject matter or materials discussed in the manuscript. OBF has been invited as a lecturer by Bristol-Meyers Squibb. MB has received Grant/Research Support from the NIH, Simons Foundation, CRC for Mental Health, Stanley Medical Research Institute, MBF, NHMRC, Beyond Blue, Geelong Medical Research Foundation, Bristol Myers Squibb, Eli Lilly, Glaxo SmithKline, Organon, Novartis, Mayne Pharma, Servier and Astra Zeneca. He has been a paid consultant for Astra Zeneca, Bristol Myers Squibb, Eli Lilly, Glaxo SmithKline, Janssen Cilag, Lundbeck and Pfizer, and a paid speaker for Astra Zeneca, Bristol Myers Squibb, Eli Lilly, Glaxo SmithKline, Janssen Cilag, Lundbeck, Organon, Pfizer, Sanofi Synthelabo, Solvay and Wyeth. AE and AL have no conflicts of interest.

## Authors' contributions

GS took part in the conception and design of the study, statistical analysis, interpretation of the findings and takes primary responsibility for writing the paper. OBF took part in the acquisition of the data, conception and design of the study, interpretation of the data and critically revised the manuscript. AE took part in the acquisition of the data, statistical analysis and critically revised the manuscript. MB took part in the interpretation of the data and critically revised the manuscript. AL took part in the conception and design of the study, acquisition of the data, interpretation of the data and critically revised the manuscript. All authors contributed to and have approved the final manuscript

## Pre-publication history

The pre-publication history for this paper can be accessed here:

http://www.biomedcentral.com/1741-7015/10/148/prepub
